# A Human β-Defensin-Based Recombinant Protein DF_2_-HSA Ameliorates Cytokine Storm

**DOI:** 10.3390/cells15020202

**Published:** 2026-01-21

**Authors:** Yibo Du, Zhuojun Yu, Weijin Sheng, Yi Li, Lei Hou, Yanbo Zheng, Xiujun Liu, Yongsu Zhen

**Affiliations:** 1 Department of Oncology, Institute of Medicinal Biotechnology, Chinese Academy of Medical Sciences and Peking Union Medical College, Beijing 100050, China; 2 Experimental Animal Center, Institute of Medicinal Biotechnology, Chinese Academy of Medical Sciences and Peking Union Medical College, Beijing 100050, China; 3 State Key Laboratory of Bioactive Substance and Function of Natural Medicines, Institute of Medicinal Biotechnology, Chinese Academy of Medical Sciences and Peking Union Medical College, Beijing 100050, China

**Keywords:** cytokine storm, DF_2_-HSA, recombinant protein, β-defensin 2, albumin

## Abstract

Cytokine storm is a critical driver of acute respiratory distress syndrome and multiple organ failure. Human β-defensin 2 (HBD-2) is the first inducible defensin discovered in human body. Defensin can resist pathogenic microorganisms invading the body through direct bactericidal effect and also modulates acquired immune response. Albumin exhibits immunomodulatory properties and can reduce the level of inflammatory cytokines to improve the systemic inflammatory response. We previously engineered a recombinant fusion protein, DF_2_-HSA, comprising two HBD-2 molecules linked to human serum albumin. Here, we evaluated its effect on cytokine storm using a lipopolysaccharide (LPS)-induced cytokine storm murine model (BALB/c athymic mice, female). DF_2_-HSA reduced the mortality in cytokine storm murine model and prolonged the retention time of HBD-2 in the body. A Luminex assay showed that DF_2_-HSA reduced the production of multiple inflammatory cytokines in cytokine storm murine model. Evans blue staining showed that DF_2_-HSA reduced vascular leakage. Transmission electron microscopy showed that DF_2_-HSA reduced the lung injury of cytokine storm mice. The pathological results showed that DF_2_-HSA alleviated the lung and small intestine damage of cytokine storm mice. In summary, DF_2_-HSA effectively inhibits cytokine storms and ameliorates associated tissue damage.

## 1. Introduction

A variety of pathogens, autoimmune conditions, malignancies, genetic disorders, and certain therapeutic interventions can trigger life-threatening systemic inflammatory syndromes. These conditions share a common pathway: an excessive immune cell activation leading to a massive dysregulated release of cytokines. This results in a self-reinforcing inflammatory feedback loop that becomes life-threatening. Such syndromes are collectively termed cytokine release syndrome (CRS), with the most severe manifestation known as a cytokine storm (CS) [1]. CS is a systemic inflammatory response mediated by cytokines [2]. It arises in contexts such as anaphylaxis, graft-versus-host disease, acute respiratory distress syndrome (ARDS), systemic inflammatory response syndrome, chimeric antigen receptor-T cell therapy, and sepsis, with the lattermost responsible for up to 19.7% of globe death [3]. The condition is defined by the dynamics and quantities of cytokine release, which inflict severe host damage [2]. Without timely recognition and adequate intervention, CS can progress from non-specific symptoms to multi-organ failure [4]. Using existing drugs (antibodies targeting interleukin-6, TNF, interferon gamma, or interleukin-1β) to neutralize the elevated levels of specific cytokines in circulation is not always effective. All targeted drugs have the risk of targeting a specific target, and combination treatment carries more potential risks than monotherapy [2]. The development of new drugs to treat CS is urgent.

Human β-defensin 2 (HBD-2) is a 64-amino acid, cysteine-rich cationic peptide with low molecular weight [5]. Like other defensins, its structure features β-sheet folds stabilized by three disulfide bonds, which are essential for maintaining the protein’s structural integrity [6]. This protein is secreted by various epithelial cells, primarily in response to microbial contact or pro-inflammatory cytokines [5]. In addition to its established antibacterial function and significance in epithelial tissues, HBD-2 also contributes to immune cell chemotaxis and activates toll-like receptors (TLR) on their surface [7]. HBD-2 is an important defensive component in the oral cavity, produced by the epithelial cells of the gum mucosa; a finding confirmed by the detection of its mRNA in these cells and its protein product in saliva [8]. In vitro studies demonstrated that synthetic HBD-2 exhibited dose-dependent antimicrobial activity against H. pylori cell suspensions, achieving complete bacterial eradication at a concentration of 10^−5^ M [9]. Recent in vitro studies indicate that HBD-2 enhances intestinal epithelial integrity by improving tight junctions between cells, thereby strengthening protection against pathogen invasion, such as the dissemination of *Candida albicans* from the gut [10]. These findings highlight HBD-2 as a promising therapeutic candidate for a range of human diseases.

Human serum albumin (HSA) is an opulent, non-glycosylated, versatile carrier protein in plasma that possesses multiple functions [11]. As a natural transporter in the blood, HSA exhibits the ability to reversibly bind to diverse endogenous and exogenous compounds, including hormones, fatty acids, and metal ions, facilitating their delivery to target tissues. This inherent binding capacity makes HSA an attractive drug carrier. Widespread advances in nanotechnology have also facilitated the development of drug delivery systems [12]. The circulatory half-life of albumin in human is 3 weeks. This property can be leveraged to extend the circulatory half-life of rapidly cleared biologics [13].

In a previous study, we designed a novel recombinant fusion protein, DF_2_-HSA, which conjugates two HBD-2 molecules to one HSA molecule [14]. In the present study, we investigate the therapeutic potential of DF_2_-HSA against CS using a lipopolysaccharide (LPS)-induced cytokine storm murine model. This model replicates the rapid cytokine release and end-organ damage characteristic of human hyperinflammatory syndromes. We hypothesize that the HBD-2 moiety will act as the primary effector to directly mitigate the dysregulated immune response, while the HSA component will serve as a carrier to significantly prolong systemic circulation. This dual mechanism will synergistically enhance the therapeutic efficacy against cytokine storm.

## 2. Materials and Methods

### 2.1. Construction of Cytokine Storm Model (CSM) Mouse

In vivo experiments were conducted under a protocol (IMB20220221D7) approved by the Animal Experiment Ethics Committee of the Institute of Medical Biotechnology, Chinese Academy of Medical Sciences. BALB/c athymic mice (6–8 weeks, 18–22 g, female) were obtained from Beijing HFK Bioscience Co. Ltd. (Beijing, China). Upon arrival, the mice were acclimated (5 per cage) in our institution’s specific pathogen-free facility for one week, under a 12 h light/dark cycle. After this period, healthy mice (no signs of significant distress, injury, or illness) were used to construct the cytokine storm model by intraperitoneal injection of LPS according to the previously reported method with some adjustments [15,16]. The mice were randomly divided into different groups, including control, model, and treatment groups, each of which contained 10 mice to yield reliable survival statistics with the fewest animals necessary. Thirty mg/kg DF_2_-HSA was administered via tail vein injection 24 h before molding, after modeling, the survival curves of mice within two weeks were recorded, and the restricted average survival time of each group was calculated. The dosage of DF_2_-HSA (30 mg/kg) was selected based on our previous report [14]. Cages belonging to different groups were evenly distributed and regularly rotated throughout the study periods. Predefined humane endpoints were strictly followed, including significant weight loss (>20%), severe lethargy, labored breathing, or inability to eat/drink. There were no exclusions of entire animals from the primary survival analysis. At the end of the experiment, the mice were euthanized by cervical dislocation under deep isoflurane anesthesia. Samples were processed and analyzed blindly. Statistical evaluation was performed prior to unblinding the group allocations.

### 2.2. Evaluation of Biodistribution with In Vivo Imaging

To visually observe the biological distribution and residence time of recombinant protein DF_2_-HSA in cytokine storm mice, we labeled DF_2_-HSA according to the instructions of Cy5.5 kit (Biotyscience Technology Co., Ltd., Beijing, China). Labeled DF_2_-HSA was injected intravenously (30 mg/kg), and after 24 h, the CSM was induced by intraperitoneal injection of LPS (6 mg/kg). For imaging, the mice were anesthetized with isoflurane, and fluorescence was captured at specified intervals using an IVIS-200 system (Xenogen, Hopkinton, MA, USA), with time zero defined as the moment of DF_2_-HSA administration. After 168 h, the mice were euthanized, and the liver, heart, lung, spleen, kidney, small intestine, large intestine, pancreas, stomach, and femur were collected. The distribution of Cy5.5-DF_2_-HSA in different tissues was recorded by an IVIS-200 system. Living Image 4.5.4 software was used to analyze fluorescence signals.

### 2.3. Effects of DF_2_-HSA on Multiple Cytokines in Plasma and Alveolar Lavage Fluid of Cytokine Storm Mice Model

To study the effects of DF_2_-HSA on mouse cytokines, we used a Luminex assay to detect the changes in multiple cytokines in blood and alveolar lavage fluid of mice (4 mice/group). DF_2_-HSA was pre-administered once intravenously (30 mg/kg). Twenty-four hours later, the mice in the model and treatment groups received an intraperitoneal injection of LPS (6 mg/kg) to induce CS. Following collection into anticoagulant tubes, blood was processed by centrifugation (1000× *g*, 30 min, 4 °C) to obtain plasma. The alveolar lavage solution was centrifuged at 300× *g* for 10 min at 4 °C. The supernatant was harvested and centrifuged twice. The obtained supernatant is the test sample. Mouse cytokine/chemokine profiling was conducted using a Luminex assay kit (BIO-RAD, Hercules, CA, USA) by LabEx (Shanghai, China) according to the manufacturer’s protocol. Briefly, the sample magnetic bead co-incubation, plate washing, antibody incubation, plate washing, Streptavidin-PE incubation, plate washing, and magnetic bead re-suspension were carried out, and a Luminex instrument was used to read the plate and generate data reports.

### 2.4. Effects of DF_2_-HSA on Vascular Permeability in Cytokine Storm Mice Model

To evaluate vascular permeability, Evans blue staining was used. DF_2_-HSA was pre-administered (30 mg/kg, i.v.), and 24 h later, CS was induced with LPS (6 mg/kg, i.p.). Sterile saline was used to prepare 1% Evans blue stain solution. Six hours after the mouse modeling, Evans blue dye (10 mg/kg) was injected into the tail vein, and the blood vessels of the mice turned to ink blue. The mice were killed 1 h after staining. The abdominal skin of the mice was cut with surgical scissors to expose the peritoneum. A needle syringe was used to pierce the subperitoneum and 1 mL of normal saline was administered into the abdominal cavity. The abdomen of the mice was gently kneaded for 1 min to make the leaking dye fully dissolved in the normal saline. The peritoneum of the mice was cut and the normal saline was recovered into the EP tube using a needleless syringe. The collected samples were centrifuged (1000× *g*, 10 min, 4 °C), and the supernatant was carefully transferred to a new tube. Absorbance at 620 nm was measured using a microplate reader, and the data were analyzed statistically.

### 2.5. Transmission Electron Microscopy

To observe the effect of DF_2_-HSA on the subcellular structure of mouse lung tissue in cytokine storm mice, the lung tissue of mice was observed by transmission electron microscopy. DF_2_-HSA was pre-administered once (30 mg/kg, i.v.), and 24 h later, CS was induced with LPS (6 mg/kg, i.p.). Six hours later, mice were euthanized, and the lung tissue were harvested and fixed overnight in 2.5% glutaraldehyde. The samples were first rinsed with phosphoric acid buffer and osmium tetroxide, and then dehydrated with ethanol of different concentrations. The tissue blocks were immersed in acetone and epoxy resin mixture successively, then the epoxy resin was injected with 2% catalyst, and the samples were inserted into the embedded plate and placed in the oven for polymerization. Slices of 70 nm thickness were prepared. The samples were stained with 2% uranium acetate saturated alcohol solution for 8 min, and the images were observed under transmission electron microscopy (Hitachi JEM-1200EX, Tokyo, Japan).

### 2.6. Assessment of Lung Pathology

Hematoxylin and Eosin (H&E) and Periodic Acid–Schiff (PAS) staining were used to detect the effects of DF_2_-HSA on pathological changes in lung and intestinal tissue of cytokine storm mice. DF_2_-HSA was pre-administered once (30 mg/kg, i.v.), and 24 h later, CS was induced with LPS (6 mg/kg, i.p.). Six hours later, the mice were euthanized, and the organs were dissected and fixed in 4% paraformaldehyde. Tissue sections were stained with HE and PAS, then examined microscopically to evaluate pathological changes.

### 2.7. Statistical Analysis

Data were expressed as mean ± SD (standard deviation). The normality of distribution was assessed using the Shapiro–Wilk test. Statistical analyses were performed using GraphPad Prism 8 software. The differences between groups were assessed using Student’s *t*-test (for normally distributed data) or the Mann–Whitney U test (for non-normal data), as appropriate. Survival data were analyzed using the Log-rank test. The significance levels were expressed as * *p* < 0.05, ** *p* < 0.01, and *** *p* < 0.001, ns, not significant.

## 3. Results

### 3.1. DF_2_-HSA Improved the Survival Rate of Cytokine Storm Mice

**Figure 1:**
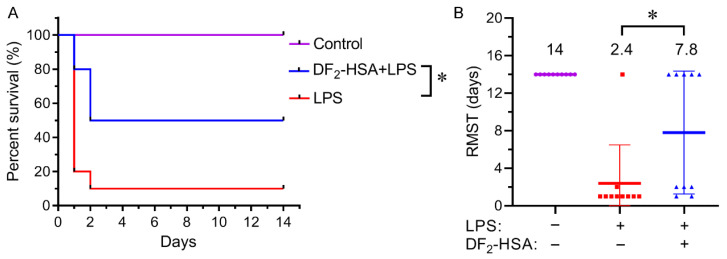
Effect of DF_2_-HSA on survivability of LPS-induced CSM mice. Survival curve (**A**), and RMST (**B**) of model mice treated with DF_2_-HSA (30 mg/kg) were analyzed. Data were expressed as the mean ± SD (*n* = 10). Log-rank test and *t*-test were used for analysis. * *p* < 0.05.

An LPS-induced CSM was used to evaluate the effect of DF_2_-HSA treatment on the survival rate of mice. A healthy control group, a model group, and a DF_2_-HSA administration group were set up. About 24 h after DF_2_-HSA administration, 6 mg/kg LPS was injected intraperitoneally to create the cytokine storm mice model. The death of mice was observed daily for 14 days starting from LPS administration. Animals still alive at the end of this period were administratively censored. The results showed that DF_2_-HSA significantly enhanced survival and reduced mortality risk in the model mice (Figure 1A). Restricted mean survival time (RMST) was calculated, with 14 days as the observation period. Compared with the LPS model group, the RMST in the DF_2_-HSA group was significantly extended, with a benefit of 5.4 days for RMST (Figure 1B).

### 3.2. DF_2_-HSA Had a Long Residence Time in Cytokine Storm Mice Model

**Figure 2:**
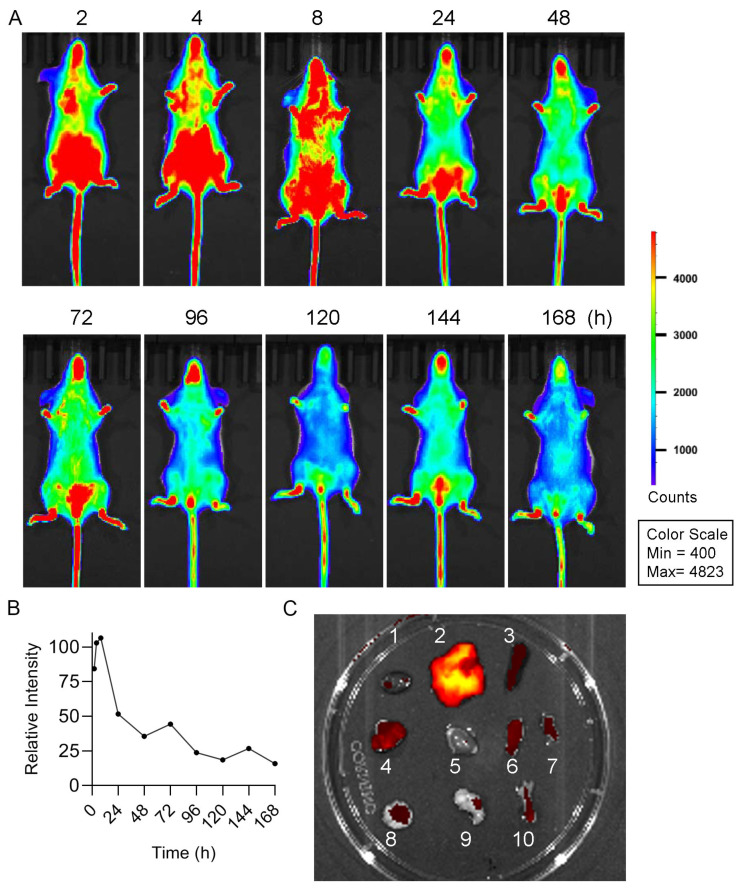
Detection of the retention time and biodistribution of DF_2_-HSA in cytokine storm model mice. (**A**) Cy5.5 labeled DF_2_-HSA (30 mg/kg) was injected into mice from tail vein, and LPS (6 mg/kg) was injected intraperitoneally 24 h later. Take the administration time of Cy5.5-DF_2_-HSA as a start, fluorescence images of Cy5.5-DF_2_-HSA were taken at different time points. (**B**) Quantification of relative fluorescence intensity of Cy5.5-DF2-HSA in mice overtime. (**C**) The distribution of Cy5.5-DF_2_-HSA in different tissues and organs ex vivo was captured after 168 h. 1−10 representing heart, liver, spleen, lung, kidney, rectum, small intestine, pancreas, stomach, and femur.

HBD-2 has good immunomodulatory function, but its molecular weight is small, its half-life is short, and it is easy to be metabolized. Previous studies of our research group showed that most of HBD-2 was metabolized out of the body after 8 h blood circulation in the mice, and almost no HBD-2 existed in the body after 72 h [14]. In order to investigate the retention time of DF_2_-HSA in cytokine storm model mice, DF_2_-HSA was labeled with Cy5.5 kit. Cy5.5-DF_2_-HSA was administered intravenously, and the retention time was observed by a small animal imaging instrument. As shown in Figure 2A, Cy5.5-DF_2_-HSA fluorescence remained detectable 168 h post-injection in cytokine storm model mice, with a signal intensity retained at approximately 18.8% of the initial level (Figure 2B). This prolonged retention demonstrates that fusion with HSA substantially extends the half-life of HBD-2, supporting its potential for sustained anti-inflammatory activity. Major organs were harvested 168 h after administration. The fluorescence intensity of different organs was detected by a small animal imaging system. As shown in Figure 2C, DF_2_-HSA was mainly distributed in liver, spleen, lung, small intestine, and large intestine of the mice. Among them, liver is the most important organ of drug metabolism with abundant blood flow. DF_2_-HSA distribution in liver may be the result of drug metabolism. The spleen has a large number of blood sinuses, which act as a filter in the blood circulation and may result in the partial retention of DF_2_-HSA. Lung, small intestine, and large intestine are abundant in epithelial cells and capillaries, while HBD-2 components of DF_2_-HSA are highly expressed in epithelial cells under physiological conditions, and HSA components are distributed in areas with high blood flow, both of which contribute to the accumulation of DF_2_-HSA in the lungs and intestines of the body.

### 3.3. DF_2_-HSA Improved Inflammation in a Cytokine Storm Mice Model

**Figure 3:**
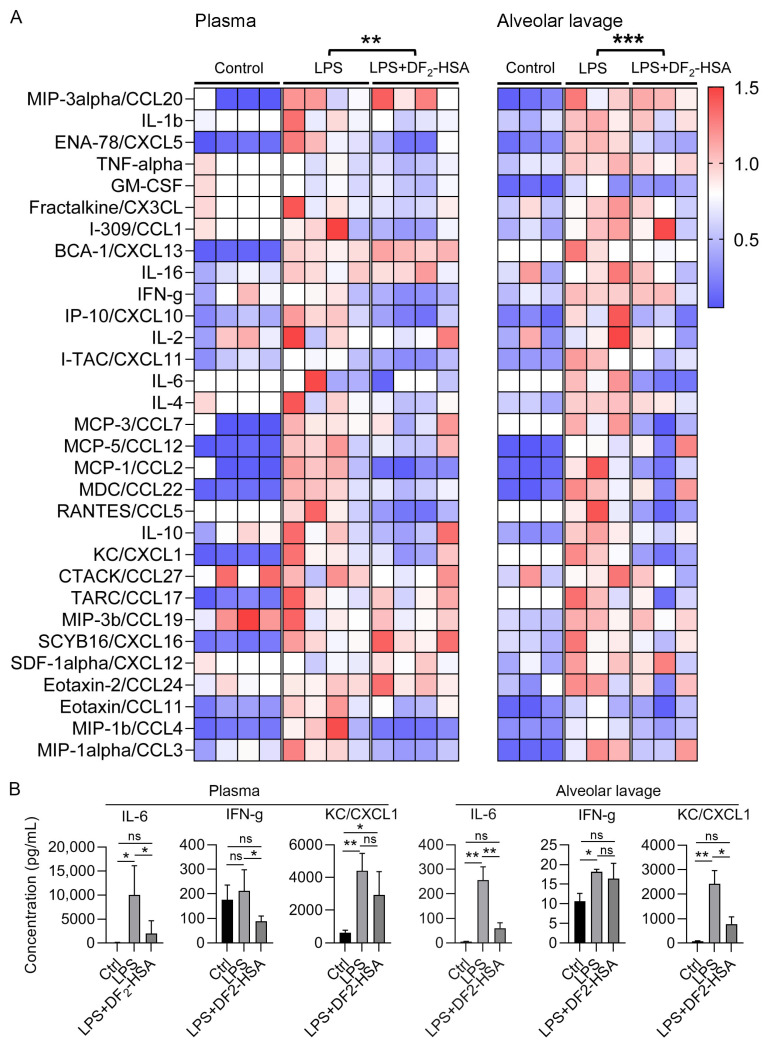
Effects of DF_2_-HSA on multiple inflammation-related factors in plasma and alveolar lavage fluid of LPS-induced cytokine storm mice. (**A**) Luminex method was used to detect 31 inflammation-related factors in plasma (*n* = 4) and alveolar lavage (*n* = 3). Heat maps showed the changes in cytokine profiles in plasma and alveolar lavage fluid of different groups of mice. (**B**). Concentration of key cytokines in the plasma and alvelar lavage. Data were expressed as mean ± SD. * *p* < 0.05, ** *p* < 0.01, *** *p* < 0.001, ns, not significant.

A Luminex assay quantified 31 cytokines and chemokines in mouse serum and alveolar lavage fluid. The concentration change index of each inflammatory factor was set as the ratio of its concentration in each group to its mean concentration in the control group. The heat map was drawn to represent the changes in the cytokine spectrum between different groups. Figure 3A showed that the levels of global inflammatory factors in serum and alveolar lavage fluid of CSM mice were significantly increased. DF_2_-HSA treatment markedly reduced these levels compared to the CSM group. Specifically, DF_2_-HSA treatment significantly reduced the concentrations of key inflammatory mediators IL-6 and IFN-g (IFN-gama) in plasma, as well as IL-6 and the neutrophil chemoattractant KC/CXCL1 in the alveolar lavage compared to the LPS model group (Figure 3B).

### 3.4. DF_2_-HSA Reduced Vascular Leakage in Cytokine Storm Model Mice

**Figure 4:**
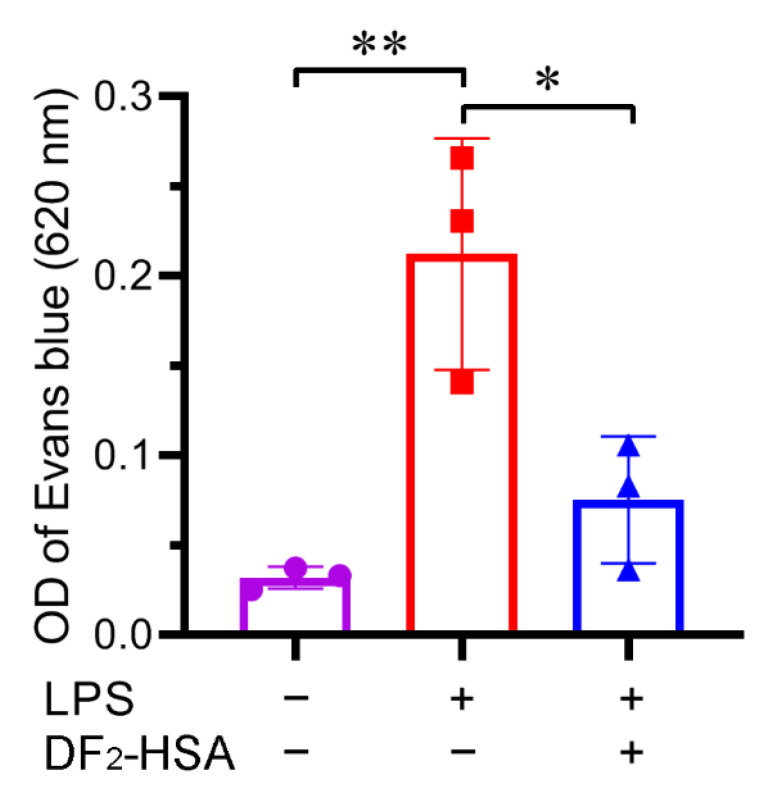
Effect of DF_2_-HSA on vascular permeability of cytokine storm model mice. The mice were treated as scheduled; 6 h later, Evans blue solution was injected through the tail vein. After 1 h, physiological saline was injected intraperitoneally to dilute the peritoneal exudate. Then the peritoneal fluid was collected and determined its OD value of 620 nm. (*n* = 3). * *p* < 0.05, ** *p* < 0.01.

Evans blue is an azo dye that binds tightly to circulating plasma proteins. Under normal physiological conditions, these protein-dye complexes are retained within the vasculature. When cytokine storm occurs, the elevated inflammatory factors cause endothelial damage and increase vascular permeability, allowing the plasma proteins to extravasate. This leakage can be quantified by measuring Evans blue accumulation in the peritoneal cavity, providing a direct assessment of vascular integrity. After 6 h of administration and modeling, Evans blue was administered intravenously to fully combine with plasma protein. One hour after staining, the mice were injected with normal saline to dissolve the peritoneal fluid. The fluid was collected and the absorbance of 620 nm was detected. As shown in Figure 4, the OD value of 620 nm of peritoneal effusion was significantly higher in the LPS model group compared to healthy controls, indicating severe vascular leakage in cytokine storm model mice. However, DF_2_-HSA treatment significantly reduced the Evans blue concentration in peritoneal effusion compared to the untreated model group, indicating that DF_2_-HSA could alleviate the vascular leakage of the cytokine storm model mice and was conducive to alleviating cytokine storm.

### 3.5. DF_2_-HSA Alleviated Lung Injury in Cytokine Storm Mice Model

**Figure 5:**
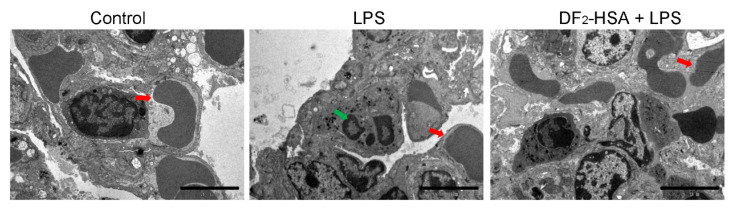
Effect of DF_2_-HSA on the ultrastructure of the lung in the CSM mice. Ultrastructural changes in lung tissues of the LPS-induced mice treated with DF_2_-HSA were observed with transmission electron microscopy. The red arrows indicate red blood cells, and the green arrows indicate infiltrating neutrophil. Scale bar = 5 μm.

Mouse lung tissue ultrastructure was examined by transmission electron microscopy (Figure 5). It was observed that the gap between red blood cells and the blood vessel wall was reduced in the lung tissue of the CSM mice, and severe congestion in the lungs was observed compared to the control mice. Furthermore, numerous neutrophils were observed in the lung tissue, indicating robust pulmonary inflammation. In contrast, the mice treated with DF_2_-HSA showed reduced symptoms of lung congestion and inflammatory cell infiltration. All these results indicate that DF_2_-HSA alleviates lung injury in the CSM mice.

### 3.6. DF_2_-HSA Improved Lung and Intestinal Tissue Pathology in Cytokine Storm Mice Model

**Figure 6:**
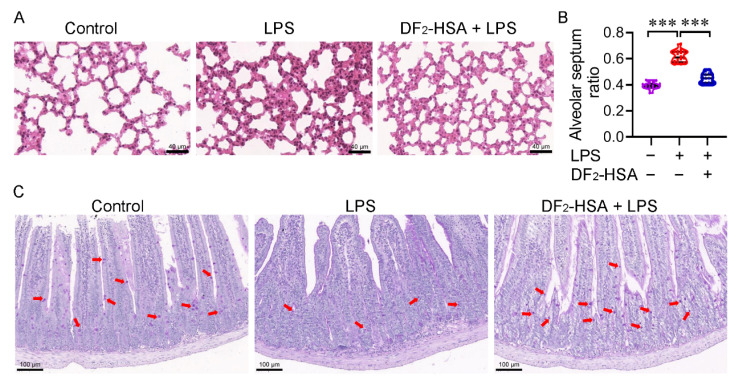
Pathological examination of the effects of DF_2_-HSA on lung and intestinal injury. CSM mice were treated with DF_2_-HSA as scheduled; then mice were sacrificed and specimens of the lung were collected. (**A**) Histopathological appearance in H&E-stained sections of lung tissues of various groups. Scale bar = 40 μm. (**B**) ImageJ 1.46r software was used to calculate alveolar septal area and total visual field area to obtain the alveolar septum/total area ratio (*n* = 16). (**C**) PAS stain was applied to stain the small intestine sections of mice in various groups. The positive staining dots indicated by the red arrows are intestinal goblet cells. Scale bar = 100 μm. *** *p* < 0.001.

Lung sections were stained with H&E. Histopathological analysis revealed a thickened pulmonary alveolar septum in the CSM mice compared to the control mice, the alveolar cavity collapsed, and the characteristics of capillary congestion and edema were presented. Lung injury was reduced in the CSM mice after DF_2_-HSA treatment (Figure 6A). A semi-quantitative analysis of mouse lung sections was performed by Image J 1.46r (Figure 6B). The alveolar septum ratio was set as the alveolar septum area divided by the total visual field area to indicate the alveolar septum thickness. Four lung tissue sections per group were analyzed. From each section, four visual fields were randomly selected along the outer edge of the lung tissue, as this region is highly responsive to inflammatory challenge and provides a representative evaluation of alveolar structure and septal thickness. As shown in Figure 6B, the alveolar septum thickened in the CSM mice induced by LPS, while the thickness of the alveolar septum decreased significantly in the CSM mice treated with DF_2_-HSA, indicating that DF_2_-HSA alleviated lung tissue congestion and edema induced by LPS-induced CSM mice. Semi-quantitative measurements corroborated the histopathological observation. Goblet cells are an important part of the intestinal barrier and are mainly responsible for the secretion of mucin, which is the primary constituent of intestinal mucus and is crucial for mucosal defense and barrier repair. Periodic Acid–Schiff staining can stain mucin and neutral mucous substances purple to indicate intestinal goblet cells. The small intestine sections of mice in different groups were stained with iodate and Schiff. As shown in Figure 6C, the number of intestinal goblet cells was obviously reduced in the LPS-treated mice compared to the control mice, and intestinal damage was severe. The number of goblet cells in the midgut was higher in the DF_2_-HSA-treated mice than in the LPS control mice, suggesting protection against intestinal damage of the model mice.

## 4. Discussion

Immune disorder [17] and vascular leakage [18] are the two main pathological features of cytokine storm. This study takes HBD-2, which has immunomodulatory effect, and HSA, which has the effect of maintaining plasma osmotic pressure, as the starting point. HSA has been widely used in the clinical treatment of hemorrhagic shock, severe burn, ARDS, acute liver failure, and other diseases [19]. A novel recombinant fusion protein DF_2_-HSA was constructed by linking two molecules of HBD-2 and one molecule of HSA through a flexible peptide segment. We obtained DF_2_-HSA by a Pichia Pastoris expression system and labeled the fusion protein with Cy5.5 fluorescent dye. In vivo imaging results revealed that DF_2_-HSA could remain in the body of model mice for more than 168 h, while in our previous study, HBD-2 was mostly metabolized within 8–12 h in the body of mice. These results indicate that the combination of HSA and HBD-2 can prolong the half-life of HBD-2 and contribute to the long-term anti-inflammatory effect.

DF_2_-HSA has both immunomodulatory and anti-vascular leakage effects. Survival analysis of the LPS-induced cytokine storm model mice showed that DF_2_-HSA significantly improved the survival rate of CSM mice and extended their restricted mean survival time. Luminex multifactor detection showed that DF_2_-HSA significantly lowered the levels of global inflammatory factors in the circulatory system of the CSM mice, indicating that DF_2_-HSA has a good immunomodulatory effect. In this study, an LPS-induced model was established. The surface of LPS has a negative charge, while the HBD-2 (DF) part of DF_2_-HSA has a positive charge, which can bind to LPS [20], block the activation of TLR4 receptor by LPS, and reduce the secretion of inflammatory factors. In addition, HSA is rich in sulfhydryl groups and is an important antioxidant in the blood [21], which can clear reactive oxygen species and reduce the oxidative stress reaction in the inflammatory site. In the Evans blue staining experiment, the model mice showed severe vascular leakage, and the related symptoms were significantly reduced in the DF_2_-HSA treatment group. Blood vessel leakage can lead to lower serum albumin levels [22]. Some researchers conducted a meta-analysis of biochemical detection indicators of nearly 3000 patients with COVID-19 [23], and found that 76% of patients had reduced serum albumin. However, decreased serum albumin levels correlated with an elevated mortality in COVID-19 patients [24]. DF_2_-HSA contains the HSA part, so that it can supplement the leaking plasma, increase the blood volume, and maintain osmotic pressure. While our in vivo data directly demonstrate DF_2_-HSA’s efficacy in reducing cytokines and vascular leakage, the proposed mechanisms of TLR4 pathway interference and oxidative stress mitigation are inferred from the established functions of its components as reported in the prior literature. Future studies at the cellular level will be essential to fully elucidate these molecular mechanisms.

DF_2_-HSA alleviated lung injury and intestinal injury in the CSM mice. The increase in plasma inflammatory factors was the most significant feature of cytokine storm, and pro-inflammatory factors can cause endothelial damage, which spreads to the whole body with blood flow, resulting in multiple organ dysfunction [25]. On the other hand, the lung [26,27] and intestine [28] were more prone to damage due to rich blood flow. Fluorescence imaging results of CSM mouse organs revealed that DF_2_-HSA showed convergent distribution in lung and intestinal tissues of the mice. HBD-2 (DF) in DF_2_-HSA is highly expressed on the epithelial surface [28], and HSA was passively targeted at the site of high blood flow [29]. Lung and intestinal tissues are rich in epithelial cells and capillaries, which are beneficial to DF_2_-HSA collection. Therefore, we focused on evaluating lung and intestinal damage in the CSM mice. The pathological evaluation of lung tissue revealed that the lungs of the CSM mice showed such pathological changes as alveolar septum thickening, alveolar collapse, congestion, and inflammatory cell infiltration, and that DF_2_-HSA treatment alleviated these changes. Analysis of BALF by Luminex revealed that DF_2_-HSA treatment significantly reduced the overall levels of 31 measured cytokines and chemokines compared to the untreated model group. All of the above indicated that DF_2_-HSA could effectively reduce lung injury in cytokine storm model mice. PAS staining revealed a significantly greater number of intestinal goblet cells in the DF_2_-HSA group compared to the model group, demonstrating a protective effect on the intestinal barrier. The intestinal tract is a congenital barrier that prevents the invasion of external pathogens [30]. Damage to the intestinal barrier not only affects the absorption of nutrients, but also causes intestinal microorganisms and toxins to enter the blood and cause sepsis [31].

It is important to note the inherent limitations of our experimental approach. The LPS-induced cytokine storm model, while robust, is driven by a single pathway (TLR4 activation) and may not fully recapitulate the complex, multifactorial triggers (e.g., viral, autoimmune, therapeutic) of human disease. Furthermore, the use of an athymic mouse model, though necessary to assess the pharmacokinetics of the human-derived DF_2_-HSA protein without immune-mediated clearance, excludes the potential role of T-cell-mediated immunity, limiting direct extrapolation to all clinical etiologies. Our study design also has two key methodological constraints: (1) the absence of control groups receiving HSA or HBD-2 alone prevented a definitive deconvolution of each component’s contribution to the observed efficacy, and (2) the prophylactic (pre-treatment) design does not model the therapeutic intervention scenario required in clinical practice. Consequently, future work in immunocompetent systems, incorporating post-symptomatic administration and comparator groups, will be critical to advance the clinical translation of DF_2_-HSA.

## 5. Conclusions

DF_2_-HSA is a novel recombinant protein, which can retain longer than HBD-2 in mice, and is conducive to play a long-term anti-inflammatory effect. DF_2_-HSA has both the immunomodulatory effect of HBD-2 and the anti-vascular leakage effect of HSA, which can significantly improve the survival rate and restricted mean survival time of cytokine storm model mice. In addition, DF_2_-HSA was convergent in the lungs and intestines of the model mice, and was shown to alleviate lung injury and intestinal injury in these mice. All of the above results indicate that DF_2_-HSA has a good anti-cytokine storm effect and has broad application prospects.

## Data Availability

Data are contained within the article.

## References

[1] Jarczak D., Nierhaus A. (2022). Cytokine Storm-Definition, Causes, and Implications. Int. J. Mol. Sci..

[2] Fajgenbaum D.C., June C.H. (2020). Cytokine Storm. N. Engl. J. Med..

[3] Rudd K.E., Johnson S.C., Agesa K.M., Shackelford K.A., Tsoi D., Kievlan D.R., Colombara D.V., Ikuta K.S., Kissoon N., Finfer S. (2020). Global, regional, and national sepsis incidence and mortality, 1990–2017: Analysis for the Global Burden of Disease Study. Lancet.

[4] Lee D.W., Gardner R., Porter D.L., Louis C.U., Ahmed N., Jensen M., Grupp S.A., Mackall C.L. (2014). Current concepts in the diagnosis and management of cytokine release syndrome. Blood.

[5] Schröder J.M., Harder J. (1999). Human beta-defensin-2. Int. J. Biochem. Cell Biol..

[6] Cieślik M., Bagińska N., Górski A., Jończyk-Matysiak E. (2021). Human β-Defensin 2 and Its Postulated Role in Modulation of the Immune Response. Cells.

[7] Seo E.S., Blaum B.S., Vargues T., De Cecco M., Deakin J.A., Lyon M., Barran P.E., Campopiano D.J., Uhrín D. (2010). Interaction of human β-defensin 2 (HBD2) with glycosaminoglycans. Biochemistry.

[8] Mathews M., Jia H.P., Guthmiller J.M., Losh G., Graham S., Johnson G.K., Tack B.F., McCray P.B. (1999). Production of beta-defensin antimicrobial peptides by the oral mucosa and salivary glands. Infect. Immun..

[9] Hamanaka Y., Nakashima M., Wada A., Ito M., Kurazono H., Hojo H., Nakahara Y., Kohno S., Hirayama T., Sekine I. (2001). Expression of human beta-defensin 2 (hBD-2) in *Helicobacter pylori* induced gastritis: Antibacterial effect of hBD-2 against *Helicobacter pylori*. Gut.

[10] Fusco A., Savio V., Donniacuo M., Perfetto B., Donnarumma G. (2021). Antimicrobial Peptides Human Beta-Defensin-2 and -3 Protect the Gut During Candida albicans Infections Enhancing the Intestinal Barrier Integrity: In Vitro Study. Front. Cell. Infect. Microbiol..

[11] Rabbani G., Ahn S.N. (2019). Structure, enzymatic activities, glycation and therapeutic potential of human serum albumin: A natural cargo. Int. J. Biol. Macromol..

[12] Li C., Zhang D., Pan Y., Chen B. (2023). Human Serum Albumin Based Nanodrug Delivery Systems: Recent Advances and Future Perspective. Polymers.

[13] Mester S., Evers M., Meyer S., Nilsen J., Greiff V., Sandlie I., Leusen J., Andersen J.T. (2021). Extended plasma half-life of albumin-binding domain fused human IgA upon pH-dependent albumin engagement of human FcRn in vitro and in vivo. mAbs.

[14] Du Y.B., Wang X.F., Liu X.J., Li Y., Miao Q.F., Jiang M., Sheng W.J., Zhen Y.S. (2022). The recombinant defensin/HSA fusion protein that inhibits NF-κb associated with intensive macropinocytosis shows potent efficacy against pancreatic cancer. Biochem. Pharmacol..

[15] Staedtke V., Bai R.Y., Kim K., Darvas M., Davila M.L., Riggins G.J., Rothman P.B., Papadopoulos N., Kinzler K.W., Vogelstein B. (2018). Disruption of a self-amplifying catecholamine loop reduces cytokine release syndrome. Nature.

[16] Yu Z., Li Y., Bai L., Zheng Y., Liu X., Zhen Y. (2024). The triple combination DBDx alleviates cytokine storm and related lung injury. Int. Immunopharmacol..

[17] Ulloa L., Tracey K.J. (2005). The “cytokine profile”: A code for sepsis. Trends Mol. Med..

[18] Opal S.M., van der Poll T. (2015). Endothelial barrier dysfunction in septic shock. J. Intern. Med..

[19] Alderson P., Bunn F., Lefebvre C., Li W.P., Li L., Roberts I., Schierhout G. (2004). Human albumin solution for resuscitation and volume expansion in critically ill patients. Cochrane Database Syst. Rev..

[20] Lee S.H., Jun H.K., Lee H.R., Chung C.P., Choi B.K. (2010). Antibacterial and lipopolysaccharide (LPS)-neutralising activity of human cationic antimicrobial peptides against periodontopathogens. Int. J. Antimicrob. Agents.

[21] Altomare A.A., Brioschi M., Eligini S., Bonomi A., Zoanni B., Iezzi A., Jemos C., Porro B., D’Alessandra Y., Guarino A. (2022). N-Acetylcysteine Regenerates In Vivo Mercaptoalbumin. Antioxidants.

[22] Matsushita S., Chuang V.T., Kanazawa M., Tanase S., Kawai K., Maruyama T., Suenaga A., Otagiri M. (2006). Recombinant human serum albumin dimer has high blood circulation activity and low vascular permeability in comparison with native human serum albumin. Pharm. Res..

[23] Rodriguez-Morales A.J., Cardona-Ospina J.A., Gutiérrez-Ocampo E., Villamizar-Peña R., Holguin-Rivera Y., Escalera-Antezana J.P., Alvarado-Arnez L.E., Bonilla-Aldana D.K., Franco-Paredes C., Henao-Martinez A.F. (2020). Clinical, laboratory and imaging features of COVID-19: A systematic review and meta-analysis. Travel Med. Infect. Dis..

[24] Violi F., Cangemi R., Romiti G.F., Ceccarelli G., Oliva A., Alessandri F., Pirro M., Pignatelli P., Lichtner M., Carraro A. (2021). Is Albumin Predictor of Mortality in COVID-19? Antioxid. Redox Signal..

[25] Wang H., Ma S. (2008). The cytokine storm and factors determining the sequence and severity of organ dysfunction in multiple organ dysfunction syndrome. Am. J. Emerg. Med..

[26] Villar J., Zhang H., Slutsky A.S. (2019). Lung Repair and Regeneration in ARDS: Role of PECAM1 and Wnt Signaling. Chest.

[27] Wang C., Xie J., Zhao L., Fei X., Zhang H., Tan Y., Nie X., Zhou L., Liu Z., Ren Y. (2020). Alveolar macrophage dysfunction and cytokine storm in the pathogenesis of two severe COVID-19 patients. eBioMedicine.

[28] Wu X., Jing H., Wang C., Wang Y., Zuo N., Jiang T., Novakovic V.A., Shi J. (2022). Intestinal Damage in COVID-19: SARS-CoV-2 Infection and Intestinal Thrombosis. Front. Microbiol..

[29] Cho H., Jeon S.I., Ahn C.H., Shim M.K., Kim K. (2022). Emerging Albumin-Binding Anticancer Drugs for Tumor-Targeted Drug Delivery: Current Understandings and Clinical Translation. Pharmaceutics.

[30] Minton K. (2022). Intestinal barrier protection. Nat. Rev. Immunol..

[31] Hu Q., Ren H., Li G., Wang D., Zhou Q., Wu J., Zheng J., Huang J., Slade D.A., Wu X. (2019). STING-mediated intestinal barrier dysfunction contributes to lethal sepsis. eBioMedicine.

